# Programming cancer through phase-functionalized silicon based biomaterials

**DOI:** 10.1038/srep10826

**Published:** 2015-06-04

**Authors:** Priyatha Premnath, Krishnan Venkatakrishnan, Bo Tan

**Affiliations:** 1 Department of Mechanical and Industrial Engineering, Ryerson University, 350 Victoria Street, Toronto M5B2K3; 2 Department of Aerospace Engineering, Ryerson University, 350 Victoria Street, Toronto M5B2K3.

## Abstract

Applications of biomaterials in cancer therapy has been limited to drug delivery systems and markers in radiation therapy. In this article, we introduce the concept of phase-functionalization of silicon to preferentially select cancer cell populations for survival in a catalyst and additive free approach. Silicon is phase-functionalized by the interaction of ultrafast laser pulses, resulting in the formation of rare phases of SiO_2_ in conjunction with differing silicon crystal lattices. The degree of phase-functionalization is programmed to dictate the degree of repulsion of cancer cells. Unstable phases of silicon oxides are synthesized during phase-functionalization and remain stable at ambient conditions. This change in phase of silicon as well as formation of oxides contributes to changes in surface chemistry as well as surface energy. These material properties elicit in precise control of migration, cytoskeleton shape, direction and population. To the best of our knowledge, phase-functionalized silicon without any changes in topology or additive layers and its applications in cancer therapy has not been reported before. This unique programmable phase-functionalized silicon has the potential to change current trends in cancer research and generate focus on biomaterials as cancer repelling or potentially cancer killing surfaces.

Research in cancer treatment is geared towards targeted therapy, gene therapy, radiation therapy, photodynamic therapy and biological therapies^1^,^2^,^3^,^4^,^5^. While the use of biomaterials for cancer therapies is envisaged, its usage has been limited as drug carriers. We foresee the use of functionalized biomaterials as surfaces that can potentially be enforced to prevent the recurrence of cancer. Likewise, research in detection of cancer is also carried out. This research primarily focuses on the use of microfluidic channels to detect the presence of cancer, often using proteins specific to the cancer cells^6^,^7^. This application requires a material that is capable of promoting the directional flow of cells as well as trapping singular cells for analysis.

Biomaterials in cancer treatments arose predominantly in the area of imaging, drug delivery and targeted therapy. Purushotham *et al.* implemented magnetite coated polymer core shell materials to release anti-cancer drugs^8^. This core shell material is temperature dependent and is therefore able to selectively release the drug over time. Several such nanoparticles are being investigated as possible candidates for drug delivery systems. However, the toxicity of the nanoparticles itself is a concern. For instance, Singh *et al.* performed an in-depth review of the toxic effects of iron oxide nanoparticles^9^. It is observed that size as well as concentration has an important role in cytotoxicity. Cancer imaging also implements similar magnetic nanoparticles for guided imaging^10^.Targeted delivery uses nanoparticles as their primary carriers coated with specific antibodies to target precise moieties. Bae *et al.* studied the applicability of manganese oxide nanoparticles in targeted siRNA (small interfering RNA) delivery as well as magnetic resonance imaging^11^.Silicon is being considered increasingly for cancer therapies. Silicon is used in various forms-crystalline, amorphous, nanoparticles and nanowires^12^,^13^,^14^,^15^. Silicon is a viable candidate owing to its biocompatibility and biodegradability. The predominance of silicon in cancer therapies has been limited to nano and micro particles. It is apparent that bulk silicon has not been investigated for its potential anti-cancer properties.

While cancer treatment therapies have focussed on nano and micro forms of silicon, cancer diagnostics essentially focuses on bulk silicon as a base. Diagnostic devices often require significant changes to their surface to suit applications. To this end, researchers rely on several techniques to change the properties of bulk silicon. These techniques can be categorized broadly as chemical and topographical techniques^16^. These techniques either change the surface topology of silicon or change its chemical characteristics. Moreover, to change material properties, these techniques occasionally require the inclusion of several additional layers. Modifications to the surface of silicon are required to allow the precisely controlled growth of cells. Characteristics such as cell adhesion, direction and single cell isolation necessitate modifications. These properties are specifically of prime importance in micro-fluidic and diagnostic devices. For example, Chen *et al.* reviewed topography modifications to capture cancer cells^17^. Micro pillars were fabricated on the surface to enhance the interactions of cells with the underlying substrate. Such topographical modifications are not only expensive but also limited in their feature size. With respect to modifications based on chemical changes, the introduction of unknown toxins in a biological environment is a possibility. Consequently, there is need for a technique that negates the use of chemicals as well as prevents changes to the topography.

In this article, we introduce the phase functionalization of silicon, converting it from a biocompatible surface to a cancer controlling surface. Ultrafast laser pulses with nano to microsecond pulse to pulse separation time interact with silicon, modifying its surface without affecting topography. This interaction can be precisely manipulated to induce cell controlling zones by creating changes in surface chemistry. The controlling zones can direct and channel cell migration as well as promote single cell channelling and isolation. Further, the phase-functionalized surface can completely repel cells from its surface via formation of oxide as well as varied crystal structures. These unique properties are attributed to the modified silicon surface. Material characterization demonstrates the presence of polymorphs of silicon dioxides such as tridymite, coesite and stishovite. The involvement of high temperature and pressure in the formation of these polymorphs allows for the existence of conventionally unstable phases at ambient temperatures. Further, the high temperature and pressure also induces changes in the crystal lattice of silicon, resulting in the generation of different phases of silicon. In this research paper we elucidate the synthesis and characterization of this phase-functionalized biomaterial. Moreover, the influence of altered surface chemistry on the growth patterns of cancer cells is explored in-depth. Figure 1 gives an overview of the process as well as applications in cancer therapies.

## Results and Discussion

### Synthesis of phase-functionalized zones

The interaction of the ultrafast pulses (micro to nano pulse separation time) and silicon results in phase-functionalized zones due to phase transformations. These phase-functionalized zones consist of different silicon and silicon oxide polymorphs as well as changes to the substrate crystal lattice. When the pulses interact with the surface of silicon, energy is transferred to the silicon surface. Due to the ultrashort nature of the pulses, multiphoton excitation processes occur. The electron temperature is now higher than the lattice temperature resulting in electron thermalization^18^. These electrons cool down and release phonons^19^. At this point the temperature of laser-material temperature evolves. It should be noted that the frequency of the pulses is an important consideration. When the required energy has accumulated, silicon converts from its solid to liquid state. Due to femtosecond regime of the pulses generates recoil pressure is generated^20^. This recoil pressures is in turn responsible for phase changes in the lattice of silicon.

In this research study, the peak power of the pulse is varied based on the frequency as well as duration of the pulses. The frequency and the width of the pulse are inversely proportional to the peak power. The surface temperature at silicon is calculated as^21^


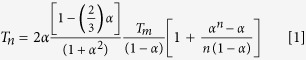


Where


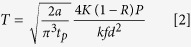


*t*_*p*_ is the pulse duration, *a* is the thermal diffusion coefficient, k is the heat conduction coefficient, R is the reflection coefficient, K is the residual energy coefficient, d is the laser spot diameter and α is a ratio of pulse duration to pulse interval.

Table 1 (Supplementary information) presents the temperature generated on the surface after multiple femtosecond pulses interact with the surface of silicon. It is evident from the temperatures that the frequency of the pulses plays a more dominant role in changing phases compared to the duration of pulses. However, the width of the phase-functionalized zone is determined by the duration of the pulses. Therefore, the frequency of pulses determines the presence or absence of the phase-functionalized zone and the pulse duration determines the width of the phase-functionalized zone.

It is observed that only at low frequency, a phase-functionalized zone is being generated. At mid frequency and high frequency, a phase-functionalized zone is not generated but the phase of the substrate is changed inducing characteristics of cells which will be delineated. To summarize, a minimum temperature of approximately 6200 K is required to generate the phase transformed zone. In spite of the similar temperatures being generated at the surface at short, mid and long pulses, the laser-material interactions are different owing to varied heat dissipation mechanisms. Changes in phases are not solely attributed to temperatures but changes in pressure generated during laser-material interaction as well. During ablation, the pressure being generated is proportional to material ablation. The formation of a trench reflects light from the sides which in turn increases the rate of ablation^22^. Subsequently, magnitude of pressure waves being generated increases. Higher pulse durations will contribute to the formation of a melt layer in this trench^23^. This may result lesser magnitudes of pressure waves. The difference in pressure waves suggests the formation of different phases of silicon as well different widths of the phase transformed zones.

### Material investigation of phase-functionalized zones

Phase-functionalized zones on silicon are observed using a scanning electron microscope (SEM) in conjunction with Energy-dispersive X-ray spectroscopy (EDX). SEM images show the presence of trenches followed by areas of phase transformation (observed by minor change in color). EDX reveals the presence of oxides on these phase transformed zones. This ratio of silicon to oxygen on the phase transformed surfaces does not directly relate to peak power. Both the duration of the pulses as well as the frequency of the pulses have an effect on oxide content. With respect to frequency of pulses, a lower frequency causes greater oxide content in the phase transformed zones (Supplementary information Figure S1 (B, C)). As the frequency of pulses increases, it is evident that the oxide content at both pulse durations decreases. Moreover, oxide content at higher frequencies for both pulse widths are essentially the same. These results confirm the dominant role of frequency of pulses. The role of these oxides are further probed to study a potential correlation to cancer repelling properties.

**Figure 2:**
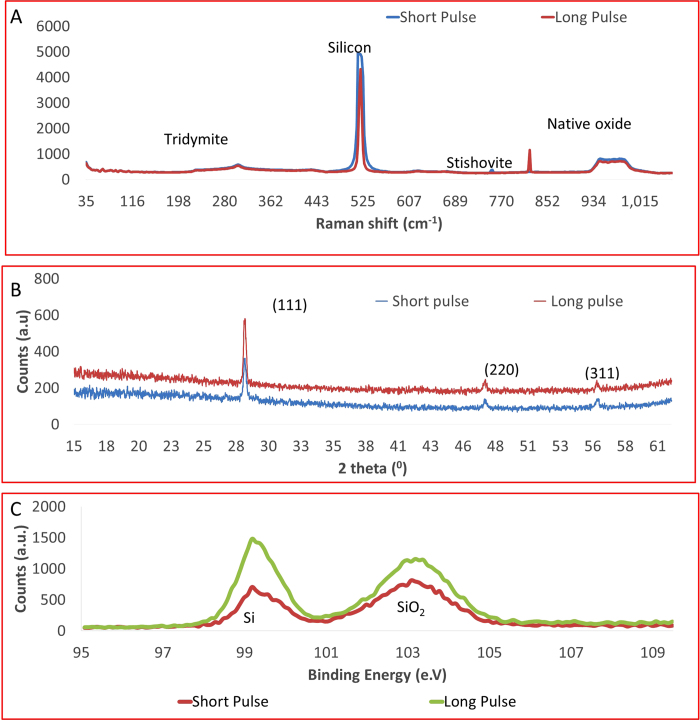
(A) micro-Raman spectra of phase-functionalized phase transformed silicon at different pulse widths (B) XRD micro spectra and (V) XPS analysis of phase-functionalized phase transformed silicon.

To study the oxides on the phase-functionalized, micro-Raman analysis is performed (Fig. 2A). The peak for silicon and native oxide layer dominate the graphs due to substrate effects. There are several small peaks across the Raman spectrum. These peaks are indicative of silicon oxides of different crystal orientations. Micro-Raman spectra show presence of coesite, tridymite and stishovite^24^,^25^,^26^. Coesite and stishovite are high temperature phases of silicon while tridymite is a low temperature phase. Coesite and stishovite are not stable at ambient temperatures. They transition into other polymorphs which includes changes to its lattice parameters and consequently become metastable at normal conditions. A broader peak for tridymite might point to presence of a stable silicon polymorph compared to other oxides. Each of the peaks in the micro-Raman graph also differ in their width. This is indicative of the presence of crystalline and amorphous phases. It is observed that at shorter pulse width, the crystalline peak of silicon is wider, pointing to the presence of amorphous silicon. Therefore, at shorter pulse widths, high temperature phases are formed. Further, shorter pulse widths also promote generation of amorphous silicon.

To quantify phase-functionalized zones, X-ray diffraction and X-ray photoelectron spectroscopy is performed. Figure 2 (B) illustrates XRD micrographs that confirm the presence of different crystal structures in the phase-functionalized zones namely (111), (220) and (311). Ultrafast laser interaction induces nucleation sites. This is followed by melting and subsequent crystallization in various orientations^27^. (111) peaks have higher intensity at long pulse compared to short pulse. This is due to highly arranged stacked nature of the (111) crystals at long pulses. Furthermore, the dominance of the (111) peak is due to its lower surface energy. It is evident that the curves show an upward trend at lower angles. These upward trends correspond to amorphous phases on the substrate. The minor shifts in peaks are attributed to instrument error. XPS micro-graphs illustrate the presence of different silicon and silicon oxides (Fig. 2C). A shift is detected in peaks around 103 eV and 99 eV. The arrangement of the bonds and the ring size also leads to a shift change. A small ring size due to smaller O-Si-O bond angles causes increase in shifts^28^.

### Cancer cell and phase-functionalized functionalized zone interaction

The growth, proliferation and adhesion characteristics of cervical cancer cells is studied on phase-functionalized zones. The formation of the phase-functionalized zone is adjacent to grooves irradiated by the laser. The areas further away from the grooves are left relatively unchanged. Further, a gradient of material change is observed as we move away from the irradiation zone. This change in material characteristic influences the growth properties of cells.

**Figure 3:**
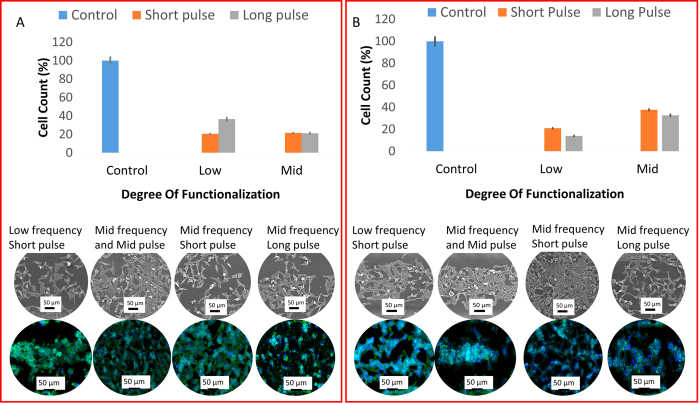
Number of cells after incubation of (A) 24 hours and (B) 48 hours. Scanning electron microscopy as well as fluorescence microscopy images showing cell growth and repelling at each phase-functionalization parameter.

Studies are performed at two time points, 24 and 48 hours. HeLa cells cultured on the surface showed interesting adhesion and growth characteristics after each time period. By varying pulse-material interaction, the cell growth is controlled. Figure 3A illustrates growth over a 24 hour period. The HeLa cells at 24 hours grow randomly and the number of cells on substrates with different parameters are similar. On average, a change in frequency of pulses did not influence the number of cells at 24 hours. However, the orientation of cells varied depending on the growth area.

At high frequency of pulses, cells are observed to adhere all over the substrate, on phase-functionalized zones as well as bulk silicon. Cells are randomly arranged and well spread out. Towards the groove however, cells stop abruptly with the majority of cells aligning themselves along the groove. At low frequency, the cells are seen growing along the line, potentially adhering well due to melt zone. At mid frequency, the cell characteristics are in between those of low and high frequency. There is no abrupt stopping at grooves and cells seem to grow over grooves. At 48 hours (Fig. 3(B)), the HeLa cells showed markedly different growth characteristics compared to the cells at 24 hours. As evidenced in Fig. 3 the cell numbers as well as cell orientation and growth properties are significantly different with respect to pulse duration as well as frequency of pulses.

**Figure 4:**
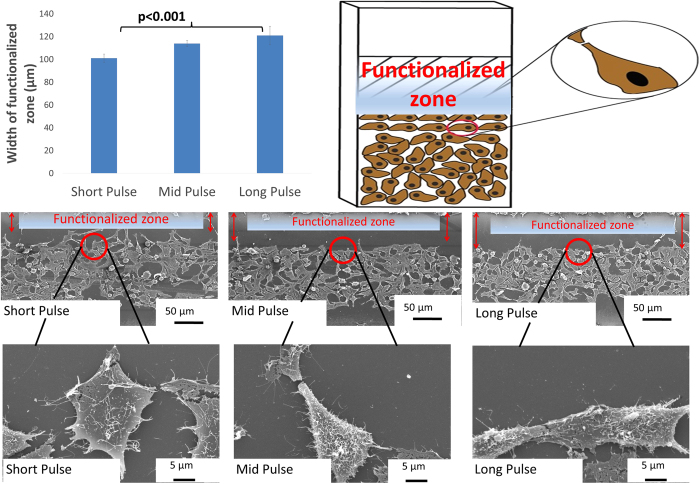
Increase in width of phase-functionalized zone with increase in duration of pulses. The SEM images are representative images at different pulse widths and the red arrows indicate phase-functionalized -transformed zones.

Low frequency consistently generated wide phase-functionalized zones at all pulse durations. At mid frequency, the cells further away from the grooves were flattened and grew randomly. The majority of cells along the grooves grew oriented to the grooves. Compared to other frequencies, it is clear that at mid frequency cells reproduced at a higher rate. The presence of a significantly higher number of rounded cells are indicative of higher cell division rates^29^. At high frequency, the number of cells that orient themselves along the groove are less. Further, cells growing away from the grooves do not attach and spread well on the surface. While frequency determined presence or absence of phase-functionalized zone, the pulse durations determined the width of these phase-functionalized zones (Fig. 4).

**Figure 5:**
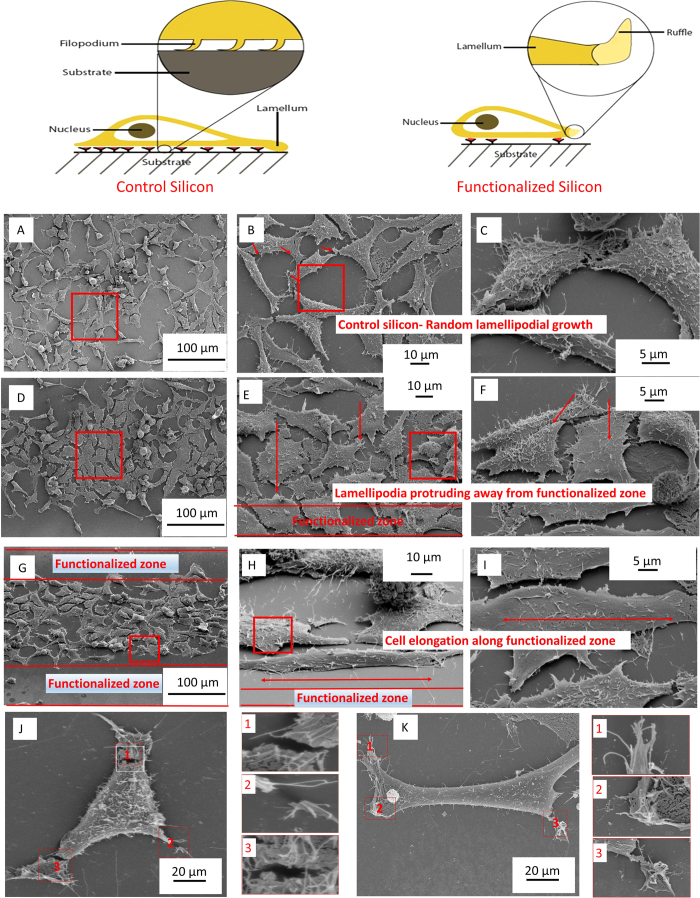
Analysis of lamellipodial and filopodial growth on control silicon as well as phase-functionalized phase transformed silicon . (**A**-**C**) HeLa cells cultured on control silicon (**D**-**E**) Hela cells cultured near phase-functionalized zones and properties of lamellipodia on such zones (**G**-**I**) HeLa cell elongation along phase-functionalized zone (**J**-**K**) The breaking up of lamellipodia close to phase-functionalized zones.

The most interesting phenomenon is the apparent activation of the phase-functionalized zone at 48 hours of incubation. Comparing Fig. 3(A) with Fig. 3 (B) at low frequency, it is evident that cells have moved away from the phase-functionalized zone. Furthermore, the cells next to the phase-functionalized zones orient themselves along the outer areas of the phase-functionalized zone. This phenomenon could be attributed to the migration of cells from one area to the other owing to changes in mechanical and chemical characteristics of the phase-functionalized zone. Figure 5 illustrates filopodial formation and protrusion away from phase-functionalized zones as opposed to random growth on control silicon surfaces. When the ultrafast pulses interact with the silicon surface, a groove and phase-functionalized zone is are generated. The heat generated during interaction dissipates laterally along the surface creating a gradient on the surface of silicon. This results in different material properties throughout this gradient. Owing to this unique feature, a trend is observed with HeLa cells after 24 and 48 hours of cell seeding.

At 24 hours even though the majority of cells accumulate away from the phase-functionalized zones, there are certain cells that tend to grow on these zones. However, at 48 hours, a clear migration of cells is observed. The HeLa cells that initially attached to the phase-functionalized zones migrate away from these zones in both directions. The unique behaviour of cells is attributed to haptotaxis^30^. Haptotaxis is movement of cells with respect to an adhesion gradient. These phase-functionalized zones create a gradient of less adhesive substrates thusly enabling the haptotaxis of cells.

**Figure 6:**
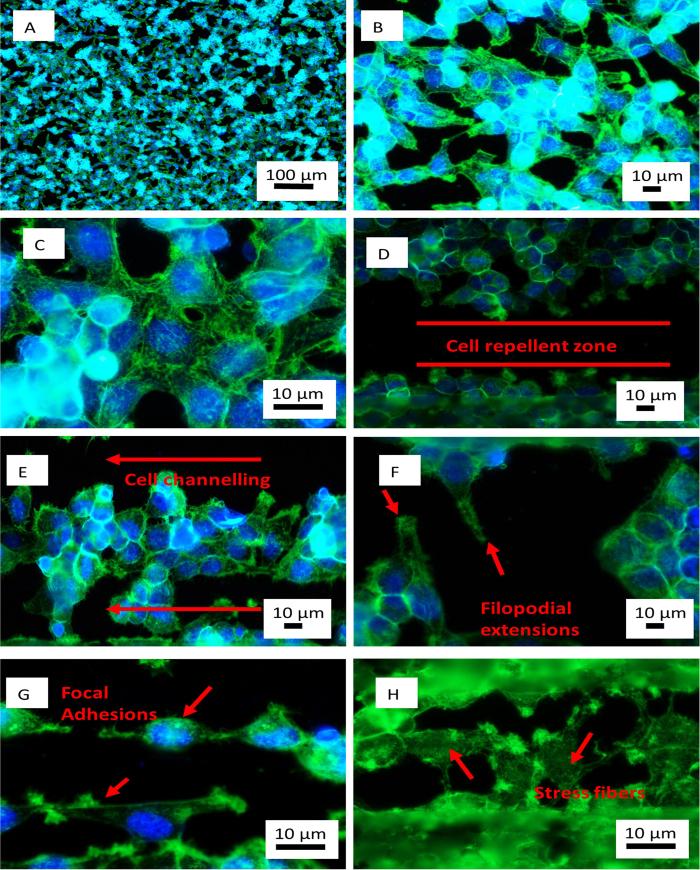
Fluorescence images of HeLa cells stained to reveal actin cytoskeleton and nucleus. (**A**-**C**) Cells growing on control silicon in a random fashion. (**D**) Low frequency and short pulse width after 48 hours of growth presenting cell repelling from phase-functionalized zones (**E**) Cell channeling, cells growing in the same direction after 48 hours, low frequency and short pulse width (**F**) Filopodial extensions sensing away from the phase-functionalized zone at high frequency and short pulse width after 24 hours of growth (**G**) Focal adhesions forming at points away from phase-functionalized zones at 48 hours, mid frequency and long pulse width (**H**) Formation of stress fibers in cells growing near phase-functionalized zones at 48 hours, mid frequency and long pulse width.

The cell adhesion and proliferation characteristics are further studied using fluorescence microscopy. The actin cytoskeleton and the nucleus of the cells are stained. Figure 6 illustrates results from fluorescence microscopy after 24 and 48 hours. Fluorescence microscopy confirmed the results obtained from SEM. Further, it also outlined the adhesion characteristics on plain silicon (Fig. 6 (A-C)) and on the phase-functionalized zones (Fig. 6(D-F)). It is observed that the cells grow in a random fashion in no particular direction on control silicon. The focal adhesion points are also randomly observed. Focal adhesions are points that interact with the substrate and aid in adhesion and migration (Fig. 6(G)).The formation of stress fibers is also observed on cells (Fig. 6 (H)). Stress fibers convert the mechanical signals from the substrate into cues for the cell^31^. The intensity and organization of stress fibers differs depending on the location of the cell. For instance, the cells growing away from the phase-functionalized zones consist of stress fibers that are not aligned in any particular direction. However, the cells growing close to the phase-functionalized zones have predominantly aligned stress fibers. Moreover, the growth of cells in areas fabricated at mid or high frequency of pulses have similarly aligned stress fibers. The stress fibers are aligned primarily along the direction of laser material interaction. Stress fibers in cells are also aligned in a direction away from the phase-functionalized zones. In all cells there is the presence of dorsal and ventral stress fibers. It has been observed that the dorsal stress fibers end in focal adhesions and this may be an indication of the leading edge of the cell. In addition to the stress fibers, the nucleus shape and polarization is also studied. Fluorescence images indicate that cells that grown on plain silicon without any substrate modifications have a well-rounded nucleus. On the other hand, the nuclei on or close to the phase-functionalized zones seem elliptical and polarized. This may be due to the passive and active forces of the actin cytoskeleton^32^. Furthermore, the filopodial extensions are seen clearly protruding in a direction away from the phase-functionalized zones.

From these results, it is evident that the formation of the phase-functionalized zones induces a significant change in material properties which in turn induces changes in the cell and phase-functionalized zone interaction. Two important interaction characteristics will be further discussed in detail in the following section.

### Cell Directionality (Cell polarization)

Phase-functionalized zone has shown that it is capable of generating cell migration in specific directions. This property is due to varying cues offered by the substrate in gradients. Owing to the adherent nature of the cells, it is apparent that contact guidance plays a vital role in the direction of migration of cells. Previous studies on directional cell migration have concentrated on substrates with topographical changes^33^. It is seen that grooves and patterned surfaces promoted directional growth. However, the phase-functionalized surfaces present no changes in topography. The phase transformation of the phase-functionalized zones take place at a sub-surface level. Therefore, the promotion of directional cell migration is attributed to presence of varying phases of silicon oxides and silicon. The formation of different phases of SiO_2_ and Si indicates varying surface energy on the phase-functionalized zones. Different surface energies further induce varying levels of wettability. Consequently, passive interaction forces act on the cells to stimulate adhesion such as Van der waals forces. However, specific adhesion required for healthy growth of cells is not achieved in these areas. As a result, cells that grow away from this region are flatter with their cytoskeleton adhering well to the substrate. While cells react to external cues to such as surface energy, internal cues also dictate the polarization of the cell. It has been postulated that cells adopt a polarized nature to sustain free minimum energy in a metastable state^34^. It is for this reason that cells grow in a polarized fashion adjacent to the phase-functionalized zone. Dickinson *et al.* stated that while quantity of adhesion ligands dictates the speed of cell locomotion, its spatial arrangement results in directional cell growth^35^. Raeber *et al.* investigated a similar trend with adherent fibroblasts and concluded that cells migrate parallel to the principle strain axis, consistent with our results^36^.

### Cancer cell repelling due to haptotaxis

Haptotaxis is the migration of cells in response to thermodynamic nature of the substrate^30^. The interaction of ultrafast pulses in the nanosecond regime with silicon results in areas of phase-functionalization with varying thermodynamic states. This change in material properties includes the introduction of stress to this substrate. This stress is then sensed by the cells itself which results in the reorganization of the stress fibers, changes in focal adhesions and hence haptotaxis of the cells away from the phase-functionalized zones. Further, a gradient of surface energies is a contributing factor. Schakenraad *et al.* stated that higher surface energies lead to higher adhesion of HeLa cells^37^. Therefore, is it is suggested that at a 48 hour period, the cells move to areas of higher surface energy, i.e. away from the phase-functionalized zones. For this reason, even though cells initially attach close to phase-functionalized zones at 24 hours, the lamellipodium is seen moving away. Another reason for initial attachment of the cells to the phase-functionalized zone may be attributed to the attraction between a positive surface and primary amine groups which are later overridden by other contributing factors. A reason why the lamellipodium is seen moving away from the phase-functionalized zones may be due to the activation of repulsion receptors^38^. It is suggested that for cells to be repelled, the actin and microtubule dynamics are changed while simultaneously reducing adhesion between cells and the substrate itself.

## Conclusion

Silicon is employed substantially in cancer diagnostics, yet its potential use as a cancer manipulative surface has not been explored. Silicon is innately a biocompatible surface and research has been focussed on its proliferative nature. We introduce the synthesis of phase-functionalized silicon generated through ultrafast laser pulse interaction. The generation of rare phases of silicon and silicon oxides and its stability at ambient temperatures is observed. This results in altered surface chemistry along a gradient which stimulates change in adhesion and migration characteristics of cancer. It is observed that the cancer cells aggressively seeks out areas away from the phase-functionalized zones. This is evidenced in the organization of stress fibers, nucleus shape as well as the actin cytoskeleton. Further, high resolution SEM images demarcate filopodial attachment and sensing away from the phase-functionalized zones. The results from this research study present significant results with precise manipulation of selective cell populations. Till date, the phase-functionalized properties of silicon have not been examined. We present a versatile technique that does not involve any additive processes or change to the topography of silicon. These unique results pave the way for a new path in cancer research. Further development of phase-functionalized silicon will provide the impetus required to change how cancer research is currently being perceived.

## Materials and Methods

### Synthesis of phase-functionalized zones

Silicon wafers are modified in a single step using ultrafast pulses with pulse separation time between micro and nanoseconds. Undoped silicon wafers <111> with a thickness of 500 μm (University Wafers, USA) are cut into 2*2 cm samples. Samples are washed with de-ionized (DI) water and ethanol and rinsed in DI water again. Consequently they are air dried. These samples are then irradiated using a diode pumped, Yb-doped femtosecond laser system (Clark-MXR Inc. IM-PULSE Series Ultrashort Pulse laser) at 4 (low frequency), 8 (mid-frequency) and 26 MHz (high frequency). The wavelength of the laser is 1040 nm. The laser irradiates the samples in an array of lines at distances between 100 μm to 2 mm. Further, different shapes to simulate tissue engineering and diagnostic device environments are synthesized. The speed at which the laser irradiates and the power of the laser is maintained at 10 mm/s and 15 W respectively. The laser pulse width or peak power plays an important role and is varied between 214 fs (short pulse), 714 fs (mid pulse) and 1428 fs (long pulse) corresponding to 8.76 MW, 2 MW and 1.31 MW respectively. All the parameters are controlled by a computer to facilitate precision and accuracy. In this paper, 4 MHz, 8 MHz and 26 MHz will be denoted as low, mid and high frequency respectively. With respect to pulse width/duration, 214 fs, 714 fs and 1428 fs will be denoted as short, mid and long pulse respectively. All samples after fabrication are ultrasonicated to remove any potential debris before cell culture is performed.

### Understanding the phase-functionalized zones

Scanning electron microscopy (SEM) (Hitachi S 5200) is performed to characterize the morphology of the phase-functionalized silicon surfaces. The samples are loaded onto aluminum stands and gold coated for SEM. Following SEM, Energy-dispersive X-ray spectroscopy (EDX) is carried out to determine the elemental composition of irradiated silicon. Micro-raman spectroscopy as well as X-Ray photon spectroscopy (Thermo scientific K-alpha) is used further to study surface composition quantitatively and qualitatively. X-ray diffraction (Siemens D5000 conventional theta/2theta diffractometer) is used as a secondary tool to confirm elemental composition.

### Cancer and phase-functionalized zone interaction

Cervical cancer cells (HeLa, ATCC, USA) are employed to qualitatively and quantitatively study cancer controllability. The cells are grown in DMEM/F12 medium supplemented with 10%fetal bovine serum and 1% pen-strep. The phase-functionalized surfaces are washed with alcohol and DI water and kept under UV light for 20 minutes. Subsequently, the substrates are placed in petri dishes and HeLa cells are seeded at a density of 10^5^ cells/ml, totalling 3 ml volume per dish. The petri dishes are placed in an incubator for 24 and 48 hours.

For SEM imaging, after the incubation period the samples are fixed in 2% glutaraldehyde in 0.1 M sodium cacodylate buffer pH 7.3 for an hour. Next, the samples are immersed in 0.1 M sodium cacodylate buffer with 0.2 M sucrose at pH 7.3 for 20 minutes. Dehydration in increasing concentrations of alcohol for 20 minutes each is followed. The samples are then critical point dried. All imaging was conducted at 5 kV and magnification was varied between 100 and 10,000 times.

For fluorescence microscopy the samples are first fixed in methanol free paraformaldehyde followed by incubation with milk to prevent non-specific binding. To stain the actin cytoskeleton, the samples are incubated with Alexa fluor 488(Life Technologies) followed by DAPI (Life Technologies) to stain the nucleus. The samples are studied using a fluorescence microscope (Nikon).

### Statistics

All experiments are carried out in triplicates and the data points are averages unless otherwise mentioned. The error bars indicate standard deviations.

## Additional Information

**How to cite this article**: Premnath, P. *et al.* Programming cancer through phase-functionalized silicon based biomaterials. *Sci. Rep.* **5**, 10826; doi: 10.1038/srep10826 (2015).

## Supplementary Material

Supplementary Information

## Figures and Tables

**Figure 1:**
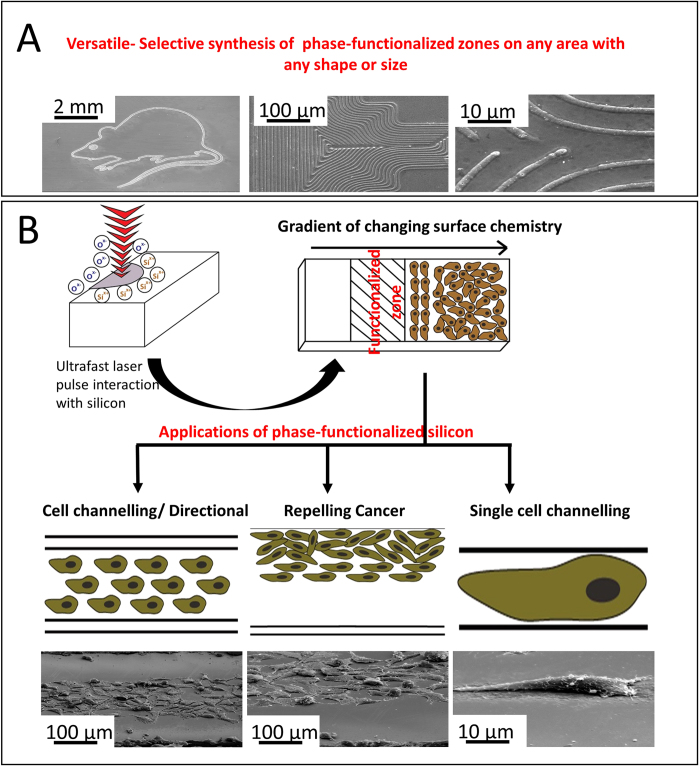
Synthesis and application of programmable phase-functionalized silicon surfaces. (**A**) The SEM images of various shapes demonstrates the versatility of the ultrafast pulse laser synthesis method. (**B**)The figure depicts an overall mechanism of fabrication and application of phase-functionalized silicon in various areas.
