# Supplementary material for: Programming cancer through phase-functionalized silicon based biomaterials
Source: Sci Rep. 2015 Jun 4;5:10826. doi: 10.1038/srep10826 (PMC4455305; doi:10.1038/srep10826)
Supplement: Supplementary Information [file srep10826-s1.doc]

**Programming cancer through phase transformed functionalized silicon based intelligent biomaterials**

Priyatha Premnath ^a^, Krishnan Venkatakrishnan ^b^*, Bo Tan ^c^

^a^ Department of Mechanical and Industrial Engineering, Ryerson University, 350 Victoria Street, Toronto M5B2K3, priyatha.premnath@ryerson.ca

^b^*Department of Mechanical and Industrial Engineering, Ryerson University, 350 Victoria Street, Toronto M5B2K3, [venkat@ryerson.ca](mailto:venkat@ryerson.ca), Phone: 416 979 5000 X6527

^c^ Department of Aerospace Engineering, Ryerson University, 350 Victoria Street, Toronto M5B2K3, [tanbo@ryerson.ca](mailto:tanbo@ryerson.ca)

| Pulse Duration (fs) | Frequency of pulses (MHz) | Peak Power (MW) | Surface temperature after multiple pulses (K) |
| --- | --- | --- | --- |
|  |  |  |  |
| 214 | 4 | 17.52 | 6242.50 |
| 214 | 8 | 8.76 | 4414.70 |
| 214 | 26 | 2.70 | 2449.70 |
| 714 | 4 | 5.25 | 6244.10 |
| 714 | 8 | 2.63 | 4416.20 |
| 714 | 26 | 0.81 | 2451.20 |
| 1428 | 4 | 2.63 | 6245.50 |
| 1428 | 8 | 1.31 | 4417.70 |
| 1428 | 26 | 0.40 | 2452.70 |

Supplementary Table 1: Surface temperature on silicon after multiple pulse interaction at various peak powers.


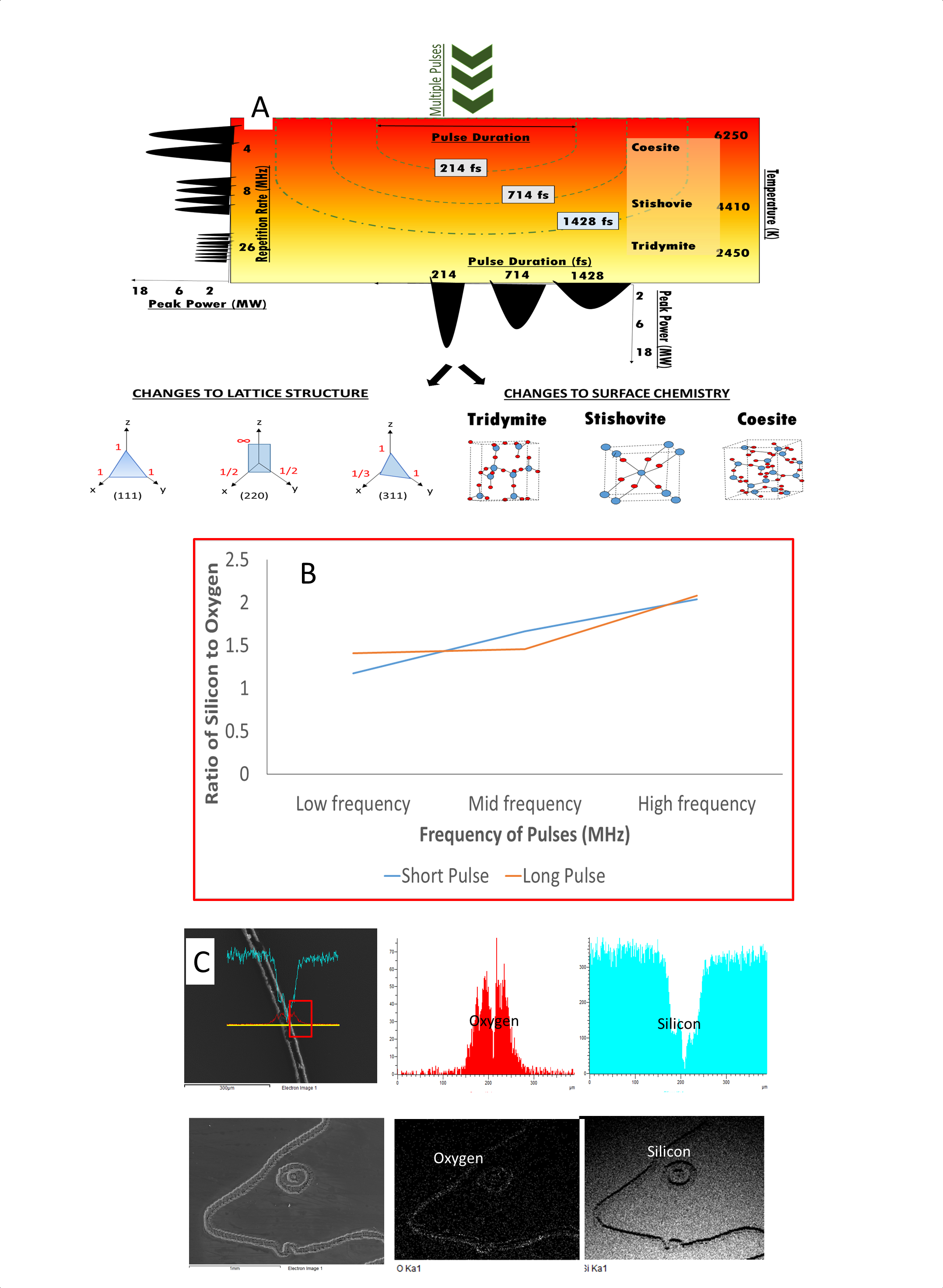


Figure S1: (A) Illustration of pulse-silicon interaction and causative changes in silicon (B) Ratio of silicon to oxygen at different pulse widths (C) EDX in mapping mode reflecting oxygen and silicon content on phase-functionalized zone
